# Siglec expression in sentinel lymph nodes in patients with oral squamous cell carcinoma

**DOI:** 10.1007/s00405-026-10276-y

**Published:** 2026-05-21

**Authors:** Lars Olaf Cardell, Eduardo I. Cardenas, Krzysztof Piersiala, Susanna Kumlien Georén

**Affiliations:** 1 Division of ENT Diseases, Department of Clinical Sciences, Intervention and Technology, Karolinska Institutet, Stockholm, Sweden; 2 Department of Otorhinolaryngology, Karolinska University Hospital, Stockholm, Sweden

**Keywords:** OSCC, Sentinel lymph nodes, Siglec receptors, Immune checkpoints, Flow cytometry

## Abstract

**Purpose:**

Siglecs (sialic acid-binding immunoglobulin-like lectins) are immune receptors that modulate leukocyte signaling and influence tumor immunosurveillance. Tumor cells exploit Siglec–sialic acid interactions via hypersialylation to dampen anti-tumor immunity. Sentinel lymph nodes (SLNs) can be conditioned by tumors to form immunosuppressive environments supporting metastasis. This study analyzes Siglec receptor expression in SLN- associated T cells from OSCC patients and evaluates correlations with tumor stage (T stage) and nodal metastasis (N stage).

**Methods:**

Sentinel lymph nodes (SLNs) were collected from OSCC patients undergoing SLN mapping/biopsy as part of standard care. Healthy lymph nodes (HLNs) were collected from non-cancer controls undergoing surgery for benign conditions. Immune cells were isolated and Siglec expression on CD4⁺ and CD8⁺ T cells were quantified using flow cytometry. Within the OSCC cohort, SLNs were classified as metastasis-negative (M⁻) or metastasis-positive (M⁺) based on histopathological assessment. Expression levels were compared between lymph node types and correlated with clinical staging parameters.

**Results:**

Distinct patterns of Siglec expression were observed between CD4 + and CD8 + T-cell subsets. Siglec-3 expression on SLN T cells was decreased in metastasis-positive (M⁺) SLNs compared with metastasis-negative (M⁻) SLNs.s. Similarly, Siglec-5 expression on T cells was decreased in SLNs from patients with higher primary tumor stage (T stage) and more extensive nodal metastasis (N stage).

**Conclusion:**

SLN immune cells exhibit distinctive Siglec expression changes associated with tumor progression. Downregulation of Siglec-3 and Siglec-5 on T cells in metastatic or advanced-stage disease, suggests that Siglec-mediated immune regulation contributes to the immunosuppressive pre-metastatic niche. These findings provide first-time evidence of altered Siglec pathways in SLNs, highlighting their potential utility as immune biomarkers and therapeutic targets in solid malignancies.

**Supplementary Information:**

The online version contains supplementary material available at 10.1007/s00405-026-10276-y.

## Introduction

Tumor cells often escape immune surveillance by exploiting inhibitory immune checkpoints in the tumor microenvironment (TME). While the PD-1/PD-L1 axis is well known, recent evidence highlights that sialic acid-binding immunoglobulin-like lectins (Siglecs) on immune cells function as glyco-immune checkpoints in cancer [1]. Siglecs are a family of leukocyte receptors that recognize sialylated glycans abundant on cell surfaces. Many Siglecs, including members of the CD33-related subfamily, contain intracellular ITIM motifs analogous to those of PD-1, recruiting SHP-1/2 phosphatases upon ligand engagement to blunt activating signals in immune cells [2]. In cancer, this Siglec–sialoglycan signaling axis is often upregulated: tumors commonly display aberrant sialylation, and tumor-associated immune cells show elevated Siglec expression, together creating an immunosuppressive loop that favors tumor immune evasion [3, 4]. Thus, the Siglec–sialoglycan axis has emerged as an important mechanism of immune suppression in the TME, akin to more established immune checkpoints such as PD-1 [5].

Among the Siglec family, Siglec-3 (CD33) and Siglec-5 are of particular interest in solid tumors. These two receptors belong to the CD33-related Siglec subset and are predominantly expressed on myeloid lineage cells (e.g. monocytes, macrophages, neutrophils, and dendritic cells) [2]. Like most inhibitory Siglecs, Siglec-3 and Siglec-5 harbor ITIM domains that transmit inhibitory signals when engaged. Functionally, their expression marks key immunosuppressive myeloid cell populations in cancer. For example, myeloid-derived suppressor cells (MDSCs)—major drivers of tumor immune evasion—are commonly identified by high CD33 (Siglec-3) expression, and these cells suppress T-cell responses and promote tumor growth by releasing arginase, nitric oxide, and other factors. Importantly, Siglec expression is not strictly restricted to the myeloid lineage: Siglec-3 (CD33) and Siglec-5 have also been reported on activated lymphoid cells, including subsets of human T cells, where they may act as inhibitory receptors [6, 7].

Notably, CD33⁺ myeloid cells infiltrate many malignancies, and their presence often correlates with worse outcomes. In oral squamous cell carcinoma (OSCC) specifically, high infiltration of CD33⁺ MDSCs in primary tumor tissues is associated with advanced T stage, lymph node metastasis, and lower survival [8]. This suggests that Siglec-3–bearing suppressor cells contribute to OSCC progression by fostering an immunosuppressive milieu that facilitates tumor spread. Siglec-5, an inhibitory receptor on neutrophils and macrophages, may play a similar role: while less studied, it can bind tumor-derived sialoglycans and transmit inhibitory signals, as seen in colon cancer models where Siglec-5 ligands dampen neutrophil activation [9]. Taken together, the evidence implicates Siglec-3, Siglec-5, and related sialic-acid-binding receptors as important modulators of anti-tumor immunity in the primary tumor microenvironment.

Sentinel lymph nodes (SLNs), the first draining lymph nodes that filter lymph from a primary tumor, are central in cancer progression and immunology. SLNs are often the initial site of metastatic spread via lymphatics, and their status is a critical prognostic factor. In OSCC and other cancers, sentinel lymph node biopsy (SLNB) is used for staging and detection of occult metastases. A positive SLN (harboring metastasis) usually warrants more extensive treatment, whereas a negative SLN spares the patient unnecessary lymph node dissection [10].

Beyond their role in metastasis, SLNs are also dynamic immunological sites. They are the first lymphoid structures to confront tumor deposits carried by dendritic cells and thus can initiate anti-tumor immune responses. However, mounting evidence indicates that tumors can “educate” sentinel nodes to become immunosuppressive even before metastasis arrives [11]. Tumor-secreted factors draining to the SLN may induce an environment rich in regulatory immune cells and tolerogenic signals. Indeed, studies have found that tumor-draining lymph nodes often exhibit an accumulation of FoxP3⁺ regulatory T cells and other immunosuppressive leukocytes, as well as increased production of immunosuppressive molecules (e.g. IL-10, TGF-β and kynurenine) by lymph node stromal cells [12]. This immunosuppressive conditioning of the SLN is thought to create a more permissive “pre-metastatic niche” for arriving cancer cells, effectively preparing the “soil” for future metastases.

Given the immunoregulatory roles of Siglec-3 and Siglec-5 in primary tumors, a question is whether these glyco-immune checkpoint mechanisms extend to the sentinel lymph nodes. To date, research on Siglec expression in SLNs is very limited. Nearly all studies of Siglecs in cancer have focused on primary tumor sites or peripheral blood; no comprehensive analysis of Siglecs in sentinel nodes has been reported. A recent study in colorectal cancer provides one of the first glimpses into this area. Siglec-15 was identified as one of the most upregulated genes in pre-metastatic sentinel nodes of colorectal cancer patients, and importantly, blocking Siglec-15 in ex vivo experiments restored T-cell activity. The authors even described their findings as “the first report” of gene-expression profiling in pre-metastatic lymph nodes, underscoring the novelty of examining Siglecs within SLNs [13]. Apart from this isolated example, no studies to date have examined Siglec-3, Siglec-5, or other Siglecs in sentinel lymph nodes in any solid tumor. This gap in knowledge is particularly pronounced in OSCC, where regional metastasis is common, but the immune characteristics of the sentinel node remain poorly understood.

Considering this gap, the aim of the present study was to investigate the expression of Siglec-3 and Siglec-5 on CD4⁺ and CD8⁺ T cells in OSCC sentinel lymph nodes, and to evaluate associations with SLN metastatic status (M⁺ vs. M⁻) and clinicopathologic stage (T stage and N stage). Healthy lymph nodes (HLNs) from non-cancer controls were included as a reference baseline for non-malignant lymph node immune phenotypes.

By characterizing Siglec profiles in SLNs, we hope to determine whether the same glyco-immune checkpoint mechanisms observed in primary tumors also operate in lymph nodes during the early steps of metastasis.

## Methods

### Patient characteristics

A total of 22 patients (14 female and 8 males, age 40–87 years), with oral cancer and 10 non-cancer patients (6 male and 4 female, age 28–53) were included in this study (see supplementary Table 1 for details). All patients were elected for tumor excision combined with a sentinel node-assisted elective neck dissection (for larger tumors), or sentinel node biopsy alone performed at Karolinska University Hospital, Stockholm, Sweden between March 3 2021 and August 14 2023. Sentinel nodes were identified with preoperative SPECT-CT and intraoperative localization using indocyanine green (ICG) fluorescence and gamma-probe confirmation. The mapping procedure was performed to define individualized drainage patterns and support pathological assessment [14, 15]. SLNs were classified as metastasis-positive (M⁺) or metastasis-negative (M⁻) based on routine histopathological assessment. Non-cancer control lymph nodes are referred to as healthy lymph nodes (HLNs) and were obtained during surgery for benign conditions; these lymph nodes were not mapped or designated as sentinel nodes. All patients were willing to participate in the study (see 2.6 Ethical approval below). Exclusion criteria were; systemic autoimmune disease, second malignancy or history of hemo-lymphopoietic malignancies or any other acute or chronic condition that could influence the immunological milieu in lymph nodes.

### Sample preparation

The unfixed neck sample and tumour samples after excision were transferred directly to the pathology department, where one of the designated pathologists handled samples and separated lymph nodes halves (all sentinel nodes and 1–2 non-sentinel nodes per patient). After surgical excision, the lymph nodes samples were kept in pre-chilled MACS Tissue Storage Solution and used within 1 h for further analysis. A Tumor Dissociation Kit (Miltenyi Biotec #130-100-008) was used to mechanically and enzymatically dissociate surgical specimens. After dissociation, cells were filtered through a 100 μm Cell Strainer (BD biosciences #352360). Finally, the samples underwent cryopreservation and were stored at -180 degrees Celsius till further analysis.

Healthy lymph nodes (HLNs) from non-cancer controls were obtained during surgeries for benign salivary gland disease (such as submandibular gland removal due to a blocked salivary duct) or neck cyst removal. These lymph nodes were processed identically to the OSCC lymph node samples but were not tracer-mapped and were not considered sentinel nodes.

### Flow cytometry

Cryopreserved single cell suspensions were thawed and first stained with Fixable Viability Stain 780 (Cat. No. 565388, BD Horizon) and blocked with Fc-block (Fc-block (Cat. No. 564220, BD Pharmingen) for 5 min at room temperature. Then samples were stained for 20 min in room temperature with surface antibodies against CD3, CD4, CD8, Siglec-3, Siglec-5, Siglec-7, Siglec-9 and Siglec-10 (Details in supplementary Table 2). The staining was followed by washing two times with PBS, 400 g, for 5 min. Cells were resuspended in 1% PFA and analysed using LRS FORTESSA (BD Bioscience). Analysis of flow cytometry data was performed with FlowJo version 10.10.0 (LLC, USA).

Cells were first gated based on side scatter (SSC-A) and forward scatter (FSC-A) to exclude debris and then live cells were gated. Then, the different Siglec-positive cells were gated on CD3^+^CD4^+^ and CD3^+^CD8^+^ cells.

#### Multiplex immunofluorescence staining (FFPE lymph nodes)

Formalin-fixed, paraffin-embedded lymph-node Sect.  (5 μm) were deparaffinized, rehydrated, and subjected to heat-induced epitope retrieval in high-pH EDTA buffer. Endogenous peroxidase was quenched, and sections were blocked prior to staining.

Multiplex labeling was performed by sequential rounds of primary antibody incubation, HRP-polymer detection, and tyramide signal amplification (TSA), followed by antibody removal before the next round. Siglec-3/5, CD4, and CD8α were stained with CF488, CF568, and CF647, respectively; nuclei were counterstained with DAPI. Panels generated were DAPI/CD4/CD8α/Siglec-3 and DAPI/CD4/CD8α/Siglec-5 (Supplier details in supplementary Table 1).

#### Imaging

Slides were mounted in Mowiol and imaged on a Zeiss Axio Scan.Z1 using a 20× objective. Four fluorescence channels (DAPI, FITC/488, 568/TRITC, 647/Cy5) were acquired as 16-bit .czi files at 0.325 μm/pixel. Representative images (~ 6.6 × 9.6 mm) were stored with a multi-resolution pyramid for downstream analysis. Multiplex immunofluorescence and whole‑slide imaging were analysed and visualised using QuPath-0.5.1 and ImageJ 1.52 h.

### Statistical analysis

Statistical analysis were performed using GraphPad Prism version 10 (GraphPad Software, La Jolla, CA, USA). Kolmogorov-Smirnov normality test was used to determine if data sets were normally distributed. A paired t test was used to compare paired groups of data, while an unpaired t test was used to compare unpaired groups of data. A p-value ≤ 0,05 was considered significant.

### Ethical approval

All procedures performed in studies involving human participants were in accordance with the ethical standards of the institutional and national research committee and with the 1964 Helsinki Declaration and its later amendments or comparable ethical standards. Informed and written consent was obtained from all individual participants included in the study. All procedures performed in this study involving human participants were approved by the Swedish Ethical Review Authority Approvals: Diary No. 2019–03518 and 2021 − 01265.

## Results

22 patients and 10 non-cancer controls were included in the study. All cancer patients had squamous cell carcinomas, the mobile tongue was the most common location (12 patients), 5 had cancer in the gum (4 lower and 1 upper), 2 in the cheek mucosa and 2 in the floor of the mouth. One SLN from each cancer patient, and one lymph node from each non-cancer patients (HLN) were analysed. Eleven (50%) OSCC patients had occult metastases in their SLN (M^+^).

### Expression of Siglec receptors on CD4⁺ and CD8⁺ T cells

**Fig. 1:**
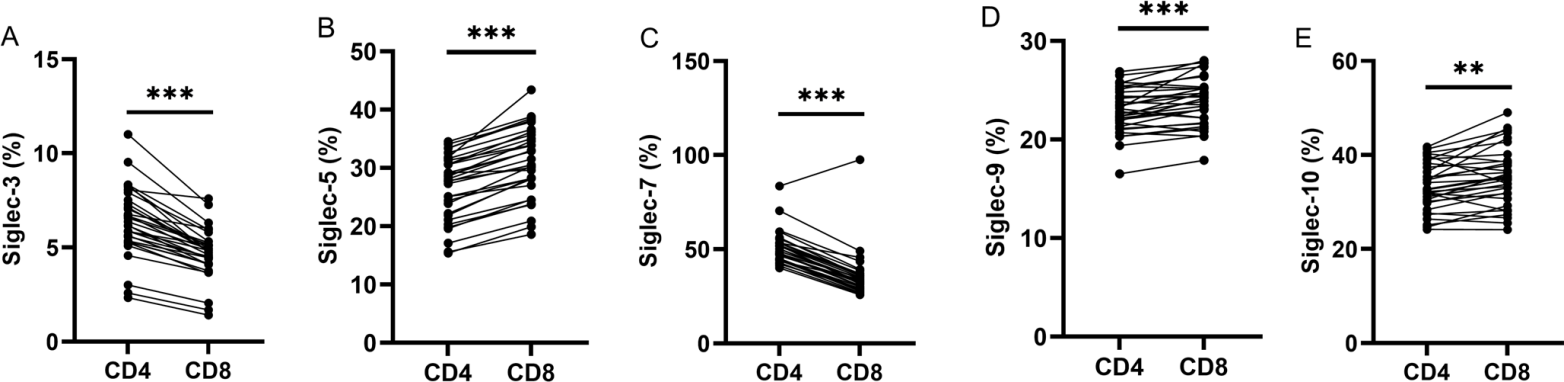
Baseline expression of inhibitory Siglecs on T-cell subsets in both Healthy and Sentinel Lymph Nodes. Flow cytometry analysis showing the distribution of Siglec-3 (**A**), Siglec-5 (**B**), Siclec-7 (**C**), Siglec-9 (**D**) and Siglec-10 (**F**) on CD4⁺ helper and CD8⁺ cytotoxic T cells. **= *p* < 0.01, ***=*p* < 0.0001 paired t test

Flow cytometry analysis confirmed that both CD4⁺ helper and CD8⁺ cytotoxic T cells expressed several inhibitory Siglecs within all lymph nodes. Siglec-3, Siglec-5, Siglec-7, Siglec-9 and Siglec-10 were detectable across both subsets, with CD8⁺ T cells showing lower expression of Siglec-3 and Siglec-7 than CD4⁺ T cells (Siglec-3: CD4 6.35 ± 0.33 vs. CD8 4.66 ± 0.24% positive cells, p < 0.001, Siglec-7: CD4 50.58 ± 1.54 vs. CD8 35.73 ± 2,22% positive cells, p < 0.001) whereas Siglec-5, Siglec-9 and Siglec-10 had higher expression in CD8+ Tcells (Siglec-5: CD4 26.65 ± 0.98 vs. CD8 30.91 ± 1.07% positive cells, p < 0.001 Siglec-9 CD4 22.94 ± 0.40 vs. CD8 23.78 ± 0.43% positive cells, p < 0.01, Siglec-10 CD4 33.32 ± 0.91 vs. CD8 35.56 ± 1.12% positive cells, p < 0.001) (Fig. 1). These baseline distributions provided the context for subsequent comparisons of Siglec expression in relation to metastatic status and tumor stage.

### Siglec-3 expression is reduced in sentinel lymph nodes harboring metastases

**Fig. 2:**
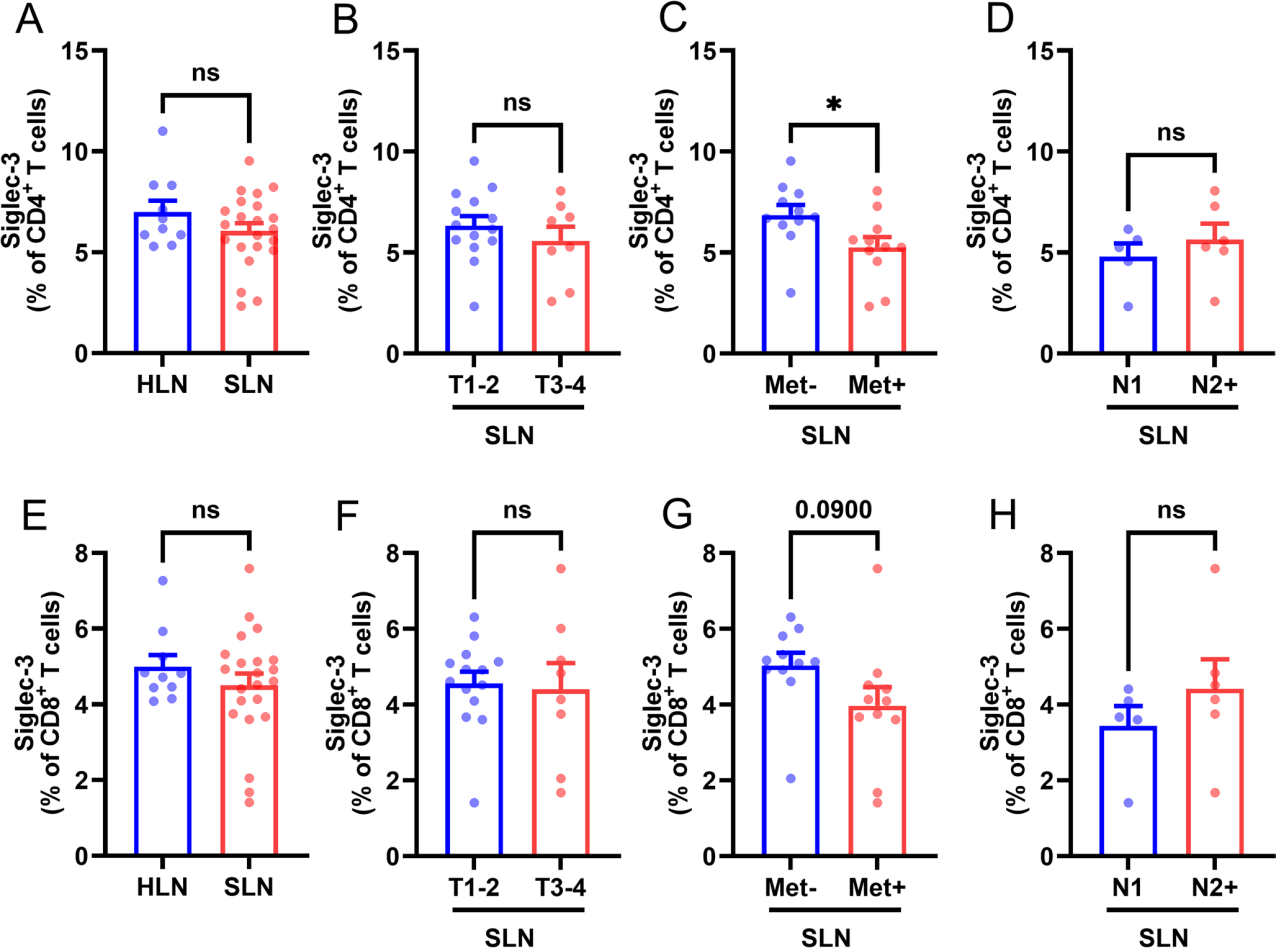
Flow cytometry data comparing Siglec-3 expression on CD4⁺ (A–D) and CD8⁺ (E–G) T cells between **A**,** E**: HLNs from non-tumour patients vs. SLNs from OSCC patients, **B**,** F**: SLNs stratified by primary tumour stage (T1–T2 vs. T3–T4), **C**,** G**: metastasis-negative (M⁻) vs. metastasis-positive (M⁺) SLNs (metastatic status defined by histopathology)and **D**,** H**: SLNs stratified by nodal stage (N1 vs. N2+). ns = not significant, *=*p* < 0.05, Unpaired T test

As shown in Fig. 2, sentinel lymph nodes with metastases (M+) displayed a reduction in Siglec-3 expression on T cells compared with metastasis-free sentinel nodes (M-). The decrease was statistically significant for CD4⁺ T cells (Fig. 2C), indicating that helper T-cell subsets are particularly affected by metastatic involvement of the sentinel node (CD4: M- 6.86 ± 0.49 vs. M + 5.26 ± 0.52% Positive cells, p < 0.05). In CD8⁺ T cells, a similar reduction was observed but did not reach statistical significance (CD8 M- 6.032 ± 0.34 vs. M + 3.97 ± 0.49% Positive cells, p = 0.09) (Fig. 2G). There was no difference in the expression of Siglec-3 between Sentinel Nodes and lymph nodes from non-tumour patients (CD4: HLN 7.00 ± 0.56 vs. SLN 6.06 ± 0.39% positive cells, non-significant (ns), CD8: HLN 5.00 ± 0.31, SLN 4.50 ± 0.31% positive cells, ns) (Fig. 2A, E), or between different T- and N-stages (CD4: T1-2 6.33 ± 0.47 vs. T3-4 5.58 ± 0.70% positive cells, ns, N1 4.79 ± 0.67 vs. N2 + 5.65 ± 0.78% positive cells, ns, CD8: T1-2 4.55 ± 0.31 vs. T3-4 4.40 ± 0.69% positive cells, ns, N1 3.44 ± 0.53 vs. N2 + 4.42 ± 0.78% positive cells, ns) (Fig. 2 B, D, E, G).

### Siglec-5 expression is associated with tumour stage and nodal involvement

**Fig. 3:**
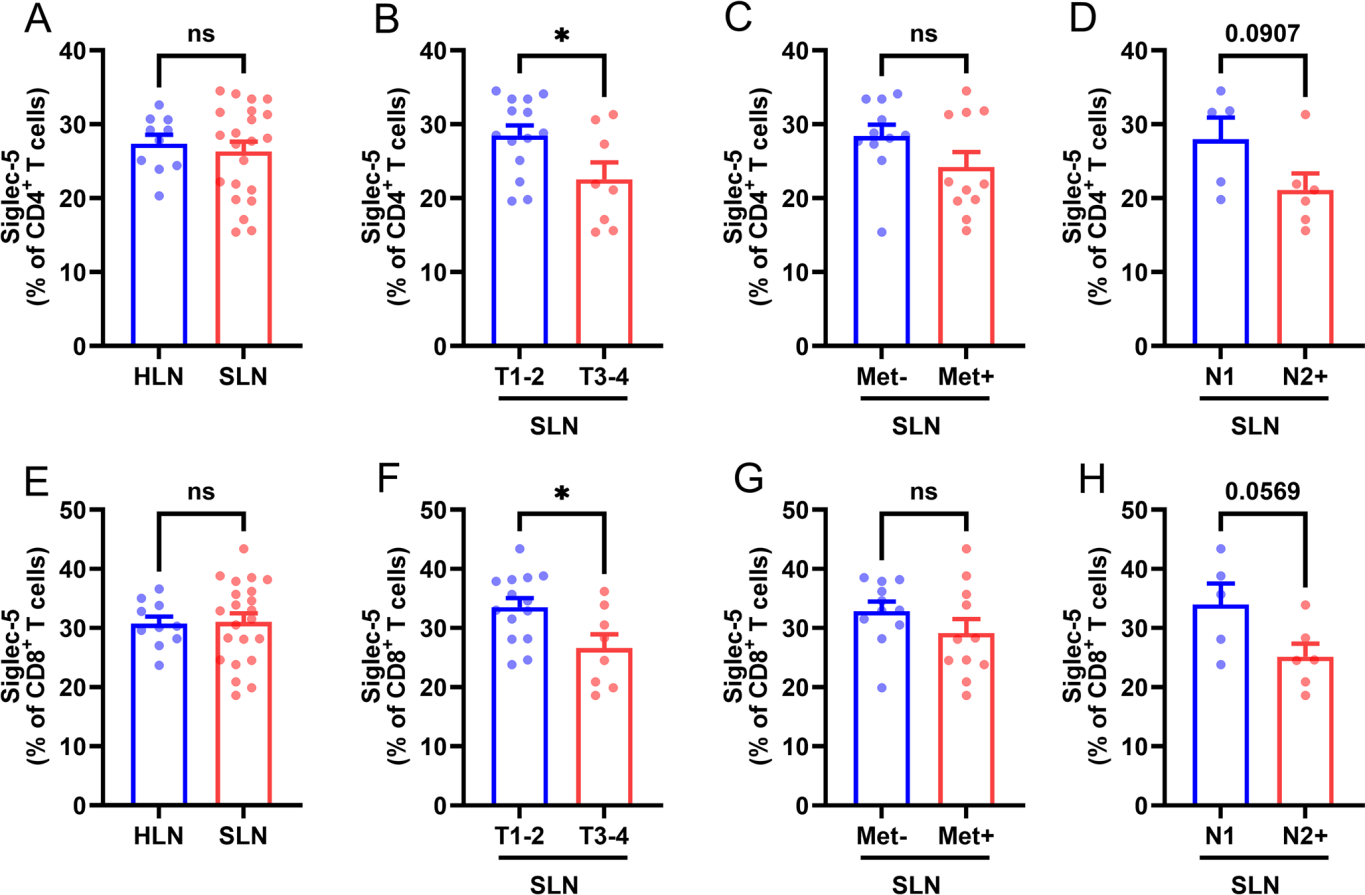
Flow cytometry data comparing Siglec-5 expression on CD4⁺ (A–D) and CD8⁺ (E–G) T cells between **A**,** E**: HLNs from non-tumour patients (HLN) vs. SLNs from OSCC patients, **B**,** F**: SLNs stratified by primary tumour stage (T1–T2 vs. T3–T4), **C**,** G**: : metastasis-negative (M⁻) vs. metastasis-positive (M⁺) SLNs (metastatic status defined by histopathology)and **D**,** H**: : SLNs stratified by nodal stage (N1 vs. N2+). ns = not significant, *=*p* < 0.05, Unpaired T test

Analysis of Siglec-5 expression revealed a distinct association with clinical stage and nodal involvement. As illustrated in Fig. 3, sentinel nodes from patients with advanced primary tumors (T3–T4) had significantly lower frequencies of Siglec-5–positive T cells compared to those from early-stage tumors (T1–T2) (CD4 T1-2 28.47 ± 1.37 vs. T3-4 22.54 ± 2.30%, p < 0.05, CD8 T1-2 33.51 ± 1.54 vs. T3-4 26.60 ± 2.32%, p < 0.05). A similar pattern was seen when stratified by nodal status: patients with more nodal metastases (N2+) displayed reduced Siglec-5 expression in sentinel nodes compared with N1 patients. These trends were observed in both CD4⁺ and CD8⁺ T-cell subsets (CD4 N1 27.98 ± 2.92 vs. N2 + 21.10 ± 2.26% p = 0.090, CD8 N1 33.96 ± 3.56 vs. N2 + 33.96 ± 2.22%, p = 0.056). By contrast, non-sentinel lymph nodes did not exhibit comparable differences, further indicating that sentinel nodes undergo distinct, tumor-driven modulation of Siglec-5 expression.

There was no relation to metastatic status and tumor stage of the expressions of Siglec-7, Siglec, 9 or Siglec-10 (results not shown).

### Localization of Siglec-3 and Siglec-5 cells within lymph nodes

**Fig. 4:**
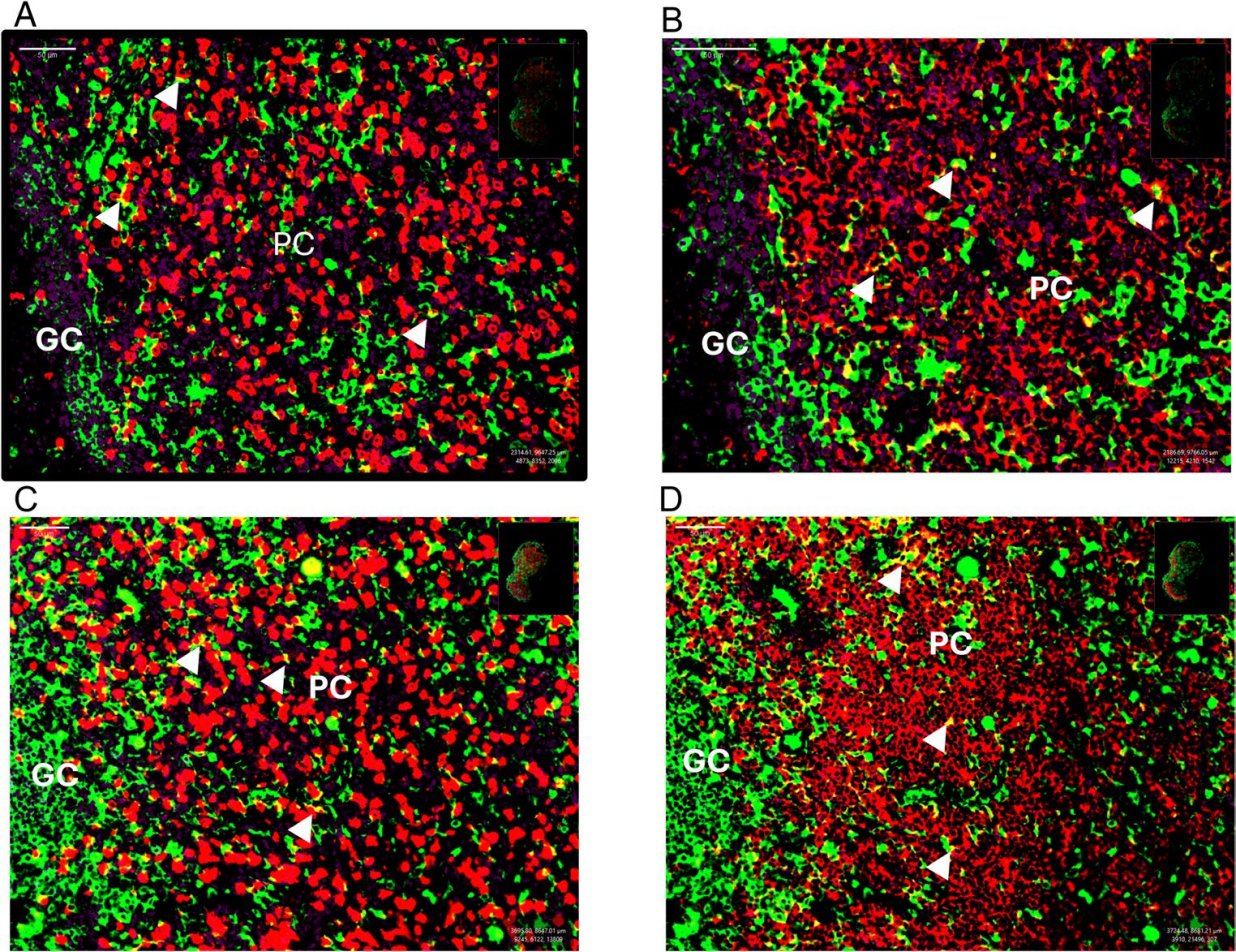
Multiplex immunofluorescence staining of Siglec expression in a metastasis-negative (M^−^), T1 SLN from a OSCC patient. Representative images of paraffin-embedded SLN sections stained for Siglec-3 and Siglec-5 together with T-cell markers. Siglec-3 (green) on CD4⁺ (red) **(A)** and CD8⁺ (red)**(B)** T cells. Siglec-5 (green) on CD4⁺ (red) **(C)** and CD8⁺ (red)**(D)** T cells. Nuclei were counterstained with DAPI (purple). Scanned images acquired using a Zeiss Axio Scan.Z1 at 20× magnification, Visualised with QuPath and Image J, in-panel labelling added in PowerPoint (365), Scalebar 50 μm. GC= Germinal Center, PC=Paracortex, Arrowhead=examples of; Siglec-3 and CD4^+^ or CD8^+^ double positive cells (**A**,** B**), and Siglec-5 and CD4^+^ or CD8^+^ double positive cells (**C**,** D**)

Multiplex immunofluorescence staining revealed that Siglec-3– and Siglec-5–positive cells were not randomly distributed within the lymph node architecture but were mainly localized to the paracortical (T-cell rich) areas (Fig. 4). In these regions, Siglec-3⁺ and Siglec-5⁺ cells were frequently observed in close proximity to CD4⁺ and CD8⁺ T cells, suggesting that these receptors may contribute to local regulation of T-cell activity within the paracortex. Few positive cells were detected in the medullary or follicular regions, indicating that their expression is largely confined to the paracortical compartment. No quantitative analysis could be made on the immunofluorescence stained slides.

## Discussion

In the present study of SLNs from OSCC patients, we identified distinctive Siglec expression profiles across T cell subsets. Helper CD4⁺ T cells and cytotoxic CD8⁺ T cells displayed varied patterns and levels of Siglec receptors, highlighting the intricate function of these immune checkpoints in modulating T cell responses in the lymph node microenvironment. Further analysis revealed that T cells from metastatic SLNs exhibited reduced expression of Siglec-3, suggesting a possible attenuation of this inhibitory pathway in the presence of metastatic disease. Additionally, the expression of Siglec-5 was found to vary in relation to tumor staging, with T cells in SLNs from patients with more advanced primary tumors (higher T stage) and extensive nodal involvement (higher N stage) showing lower levels of Siglec-5. This reduction was contrasted by higher Siglec-5 expression in T cells from SLNs of patients with early-stage tumors, indicating a potential shift in immune checkpoint dynamics as the tumor progresses and metastasizes.

Our findings demonstrate that CD4⁺ and CD8⁺ T cells in SLNs exhibit different Siglec expression profiles. This aligns with emerging evidence that, although resting human T cells minimally express Siglecs, certain Siglecs can be upregulated on specific T-cell subsets upon activation [16, 17]. In melanoma, more than 50% of intratumoral CD8⁺ T cells (but not circulating counterparts) express Siglec-9, suggesting that CD8⁺ effector T cells are particularly prone to acquiring Siglec receptors in the tumor microenvironment [18]. Consistent with these observations, we found that SLN CD8⁺ T cells tend to express higher levels of certain Siglecs than CD4⁺ T cells. This subset-skewed pattern may reflect their distinct activation and memory status, with activated effector/memory CD8⁺ T cells inducing Siglec expression as a negative feedback mechanism, whereas CD4⁺ T cells, often more naïve or regulatory in SLNs, remain largely Siglec-low [6, 8].

The differential Siglec profile suggests that CD8⁺ T cells in SLNs could be preferentially regulated by glyco-immune checkpoint interactions. Notably, Siglec-7 and Siglec-9 on CD8⁺ effector T cells can directly attenuate TCR signaling, blunting cytotoxic responses analogously to PD-1 but via sialoglycan ligands [13, 19]. Such mechanisms likely operate in SLNs as well, given the similar glycan-rich milieu. In primary tumors, enrichment of Siglec⁺ CD8⁺ T cells is associated with T-cell exhaustion and poorer outcomes [7]. Even before T cells encounter the tumor, the SLN environment, educated by tumor-derived antigens, may selectively modulate CD8⁺ vs. CD4⁺ cells via Siglec pathways, potentially influencing which T-cell subset mounts effective immunity in SLNs. Consistent with the spatial compartmentalization observed by multiplex immunofluorescence in Fig. 4, Siglec-3 and Siglec-5 staining was prominent in germinal centers and the paracortex of the tumor-draining SLN, which suggests that Siglec-mediated inhibitory signaling may influence immune activation across B-cell zones and T-cell zones. This distribution supports Siglec-3/5 as candidate biomarkers of an immunoregulatory SLN microenvironment and it motivates further investigation of the sialic acid–Siglec axis in a potential mechanism contributing to nodal immune escape and metastatic permissiveness [1, 7].

We found that SLNs harboring metastases had markedly lower Siglec-3 (CD33) expression on T cells compared to metastasis-free SLNs. Importantly, our measurements relate to Siglec-3 on T cells in the SLNs compartment and are therefore not directly comparable to studies in primary tumors where Siglec-3 is most commonly used to identify immunosuppressive myeloid populations. Nonetheless, this pattern contrasts with primary tumor settings, where high CD33/Siglec-3 is typically associated with immunosuppressive myeloid cells and advanced disease [10, 20]. In OSCC tumors, increased infiltration of CD33⁺ myeloid-derived suppressor cells (MDSCs) correlates with higher T stage, nodal metastasis, and worse prognosis [11].

The decreased Siglec-3 on T cells in metastatic SLNs may reflect an altered immune contexture due to tumor invasion. One interpretation is that metastatic conditioning of SLNs impairs local priming and activation, resulting in fewer Siglec-positive activated T cells. Tumor-induced conditioning of SLNs, via immunosuppressive cytokines such as IL-10 and TGF-β, is known to inhibit local T-cell priming, which could explain the reduction in Siglec-3. Alternatively, chronic exposure to sialoglycan ligands in the draining node could drive receptor engagement followed by internalization or shedding, yielding an apparent downregulation at the cell surface. Finally, advanced disease may alter trafficking and the balance of T-cell subsets in the SLN (e.g., fewer effector/memory cells and relatively more naïve/regulatory cells), which could secondarily reduce the frequency of Siglec-expressing T cells [2, 21].

We observed a significant decrease in Siglec-5 expression on T cells in SLNs from patients with higher primary tumor stage (T3–T4) and nodal metastases (N⁺) compared to early-stage (T1–T2, N0) patients. In primary tumor studies, Siglec-5 has been less studied than Siglec-3/9, but emerging data indicate it plays an immunoregulatory role [5]. Recent work identified Siglec-5 as an inhibitory checkpoint on human T cells that is induced upon TCR stimulation. Engagement of Siglec-5 on activated T cells (for example, by tumor-associated sialoglycans) suppresses their cytokine production and cytotoxicity [22]. Tumors can exploit this: some cancer cells express ligands for Siglec-5, enabling them to directly dampen T-cell activity via Siglec-5 interactions [7].

Given this, one might expect higher Siglec-5 on T cells in more advanced disease, where tumor immune evasion is pronounced. Paradoxically, our data show the opposite in SLNs, where advanced-stage patients had lower T-cell Siglec-5. One interpretation is that sustained engagement of Siglec-5 in earlier stages leads to subsequent internalization or shedding in late-stage disease [22, 23]. An additional, non-mutually exclusive explanation is that advanced-stage disease reduces the proportion of recently activated Siglec-5–inducible T-cell subsets in the draining node, consistent with impaired priming and/or altered composition of the SLN T-cell compartment.

Several limitations should be acknowledged. First, HLNs from non-cancer surgeries were included as a reference baseline but may differ from tumor-draining lymph nodes in OSCC due to baseline microenvironmental differences; therefore, comparisons involving HLNs should be interpreted cautiously and were not used as the primary basis for the study’s conclusions. Second, our flow cytometry analyses focused on lymph node compartments (SLNs), and matched primary tumor tissue was not available for parallel Siglec profiling; this limits direct mechanistic comparison between the primary tumor microenvironment and the draining node. Finally, extranodal extension (ENE) is an important prognostic feature of nodal disease; however, ENE was not assessed as a study variable here due to limited cohort size in the metastasis-positive subset and lack of uniformly available ENE annotation suitable for stratified analysis. Future prospective studies incorporating standardized ENE assessment are warranted.

The presented results might have some potential clinical implications. In the future, the Siglec signatures in sentinel lymph nodes could serve as valuable biomarkers for risk stratification. For instance, altered T-cell Siglec-5 levels in an SLN might predict more aggressive disease, guiding decisions on adjuvant therapy after surgery. These glyco-immune markers might complement traditional TNM staging by indicating which patients have an immunologically “permissive” node environment for metastasis. Furthermore, the Siglec pathways themselves present potential therapeutic targets. Just as monoclonal antibodies against PD-1/PD-L1 revolutionized cancer care, antibodies or glycan-mimetics that block Siglec–sialic acid interactions could reinvigorate anti-tumor immunity. Drugs targeting Siglec checkpoints are already in development; for example, an anti-Siglec-15 antibody has shown promise in preclinical models to reverse tumor-induced immune suppression. One could speculate about a therapy that, when administered to a patient with a positive SLN, disrupts the Siglec-3/Siglec-5 mediated immune dampening and thereby boosts the immune clearance of microscopic metastatic cells.

In summary, SLN immune cells display distinctive Siglec expression patterns linked to tumor progression. Our findings demonstrate that the expression of Siglec-3 and Siglec-5 on T cells is reduced in sentinel nodes from patients with metastatic or advanced-stage disease, indicating that T-cell Siglec expression is impaired in these settings rather than upregulated. These results provide the first evidence of altered Siglec pathways in SLNs and underscore their potential as immune biomarkers and therapeutic targets in solid malignancies.

## Supplementary Information

Below is the link to the electronic supplementary material.

Supplementary Material 1

## References

[1] Duan S, Paulson JC (2020) Siglecs as Immune Cell Checkpoints in Disease. Annu Rev Immunol 38:365–395 31986070 10.1146/annurev-immunol-102419-035900

[2] Macauley MS, Crocker PR, Paulson JC (2014) Siglec-mediated regulation of immune cell function in disease. Nat Rev Immunol 14(10):653–666 25234143 10.1038/nri3737 PMC4191907

[3] Murugesan G, Weigle B, Crocker PR (2021) Siglec and anti-Siglec therapies. Curr Opin Chem Biol 62:34–42 33607404 10.1016/j.cbpa.2021.01.001

[4] Büll C, den Brok MH, Adema GJ (2014) Sweet escape: sialic acids in tumor immune evasion. Biochim Biophys Acta 1846(1):238–246 25026312 10.1016/j.bbcan.2014.07.005

[5] Bärenwaldt A, Läubli H (2019) The sialoglycan-Siglec glyco-immune checkpoint - a target for improving innate and adaptive anti-cancer immunity. Expert Opin Ther Targets 23(10):839–853 31524529 10.1080/14728222.2019.1667977

[6] Hernández-Caselles T, Martínez-Esparza M, Pérez-Oliva AB, Quintanilla-Cecconi AM, García-Alonso A, Alvarez-López DM et al (2006) A study of CD33 (SIGLEC-3) antigen expression and function on activated human T and NK cells: two isoforms of CD33 are generated by alternative splicing. J Leukoc Biol 79(1):46–58 16380601 10.1189/jlb.0205096

[7] Vuchkovska A, Glanville DG, Scurti GM, Nishimura MI, White P, Ulijasz AT et al (2022) Siglec-5 is an inhibitory immune checkpoint molecule for human T cells. Immunology 166(2):238–248 35290663 10.1111/imm.13470 PMC11590682

[8] Pang X, Fan HY, Tang YL, Wang SS, Cao MX, Wang HF et al (2020) Myeloid derived suppressor cells contribute to the malignant progression of oral squamous cell carcinoma. PLoS ONE 15(2):e0229089 32092078 10.1371/journal.pone.0229089 PMC7039453

[9] van Houtum EJH, Büll C, Cornelissen LAM, Adema GJ (2021) Siglec Signaling in the Tumor Microenvironment. Front Immunol 12:790317 34966391 10.3389/fimmu.2021.790317 PMC8710542

[10] Jang SS, Davis ME, Vera DR, Lai SY, Guo TW (2023) Role of sentinel lymph node biopsy for oral squamous cell carcinoma: Current evidence and future challenges. Head Neck 45(1):251–265 36193862 10.1002/hed.27207 PMC11081060

[11] Singh R, Choi BK (2019) Siglec1-expressing subcapsular sinus macrophages provide soil for melanoma lymph node metastasis. Elife Dec 24:8e48916 10.7554/eLife.48916 PMC6930078 31872800

[12] Leong SP, Peng M, Zhou YM, Vaquerano JE, Chang JW (2002) Cytokine profiles of sentinel lymph nodes draining the primary melanoma. Ann Surg Oncol 9(1):82–87 11833497 10.1245/aso.2002.9.1.82

[13] Du H, Tang J, Li X, Wang X, Wu L, Zhang R et al (2021) Siglec-15 Is an Immune Suppressor and Potential Target for Immunotherapy in the Pre-Metastatic Lymph Node of Colorectal Cancer. Front Cell Dev Biol 9:691937 34722496 10.3389/fcell.2021.691937 PMC8548766

[14] Bark R, Kolev A, Elliot A, Piersiala K, Näsman A, Grybäck P et al (2023) Sentinel node-assisted neck dissection in advanced oral squamous cell carcinoma-A new protocol for staging and treatment. Cancer Med 12(11):12524–12534 37084007 10.1002/cam4.5966 PMC10278494

[15] Kågedal Å, Margolin G, Held C, da Silva PFN, Piersiala K, Munck-Wikland E et al (2020) A Novel Sentinel Lymph Node Approach in Oral Squamous Cell Carcinoma. Curr Pharm Des 26(31):3834–3839 32053068 10.2174/1381612826666200213100750

[16] Haas Q, Boligan KF, Jandus C, Schneider C, Simillion C, Stanczak MA et al (2019) Siglec-9 Regulates an Effector Memory CD8(+) T-cell Subset That Congregates in the Melanoma Tumor Microenvironment. Cancer Immunol Res 7(5):707–718 30988027 10.1158/2326-6066.CIR-18-0505

[17] Haas Q, Markov N, Muerner L, Rubino V, Benjak A, Haubitz M et al (2022) Siglec-7 represents a glyco-immune checkpoint for non-exhausted effector memory CD8 + T cells with high functional and metabolic capacities. Front Immunol 13:996746 36211376 10.3389/fimmu.2022.996746 PMC9540514

[18] Stanczak MA, Siddiqui SS, Trefny MP, Thommen DS, Boligan KF, von Gunten S et al (2018) Self-associated molecular patterns mediate cancer immune evasion by engaging Siglecs on T cells. J Clin Invest 128(11):4912–4923 30130255 10.1172/JCI120612 PMC6205408

[19] Cochran AJ, Huang RR, Lee J, Itakura E, Leong SP, Essner R (2006) Tumour-induced immune modulation of sentinel lymph nodes. Nat Rev Immunol 6(9):659–670 16932751 10.1038/nri1919

[20] Montalbán-Hernández K, Cantero-Cid R, Lozano-Rodríguez R, Pascual-Iglesias A, Avendaño-Ortiz J, Casalvilla-Dueñas JC et al (2021) Soluble SIGLEC5: A New Prognosis Marker in Colorectal Cancer Patients. Cancers (Basel) 13:15 10.3390/cancers13153896 PMC8345516 34359797

[21] BoutiP, Blans C, Klein B, Shome D, Nadafi R, Van Houdt M et al (2023) SIGLEC-5/14 inhibits CD11b/CD18 integrin activation and neutrophil-mediated tumor cell cytotoxicity. Int J Mol Sci 24(24):17141 10.3390/ijms242417141 38138970 10.3390/ijms242417141 PMC10742634

[22] Läubli H, Varki A (2020) Sialic acid-binding immunoglobulin-like lectins (Siglecs) detect self-associated molecular patterns to regulate immune responses. Cell Mol Life Sci 77(4):593–605 31485715 10.1007/s00018-019-03288-x PMC7942692

[23] Schauer R, Kamerling JP (2018) Exploration of the Sialic Acid World. Adv Carbohydr Chem Biochem 75:1–213 30509400 10.1016/bs.accb.2018.09.001 PMC7112061

