# Supplementary material for: Siglec expression in sentinel lymph nodes in patients with oral squamous cell carcinoma
Source: Eur Arch Otorhinolaryngol. 2026 May 21;283(7):4715–23. doi: 10.1007/s00405-026-10276-y (PMC13388428; doi:10.1007/s00405-026-10276-y)
Supplement: Supplementary file 1 — Supplementary Material 1 [file 405_2026_10276_MOESM1_ESM.docx]

Supplementary Table 1: Patient Characteristics

| **Patient** | **Sex** | **Age at surgery** | **Localisation** | **pTNM classification** | | | **Recurrence Yes/No** |
| --- | --- | --- | --- | --- | --- | --- | --- |
|  |  |  |  | **pT-stage** | **pN-stage** | **M-stage** |  |
| OSCC 1 | F | 78 | Lower gum | pT1 | pN0itc | M0 | No |
| OSCC 2 | M | 72 | Tongue | pT3 | pN2c | M0 | Yes |
| OSCC 3 | M | 55 | Tongue | pT3 | pN3b | M0 | No |
| OSCC 4 | M | 57 | Tongue | pT3 | pN0 | M0 | No |
| OSCC 5 | F | 68 | Lower gum | pT4a | pN0 | M0 | No |
| OSCC 6 | M | 61 | Tongue | pT1 | pN1 | M0 | No |
| OSCC 7 | F | 64 | Lower gum | pT4a | pN3b | M0 | Yes |
| OSCC 8 | M | 71 | Upper gum | T4a | pN0 | M0 | Yes |
| OSCC 9 | F | 71 | Cheek | pT2 | pN0 | M0 | No |
| OSCC 10 | F | 76 | Tongue | pT2 | pN2b | M0 | No |
| OSCC 11 | M | 52 | Tongue | pT2 | pN0 | M0 | No |
| OSCC 12 | M | 40 | Tongue | pT1 | pN0 | M0 | No |
| OSCC 13 | F | 57 | Tongue | pT2 | pN1 | M0 | No |
| OSCC 14 | M | 67 | Floor of the mouth | pT1mi | pN0 | M0 | No |
| OSCC 15 | F | 60 | Tongue | pT2 | pN0itc | M0 | No |
| OSCC 16 | F | 59 | Tongue | pT2 | pN0 | M0 | No |
| OSCC 17 | F | 87 | Floor of the mouth | pT2 | pN1 | M0 | No |
| OSCC 18 | F | 61 | Lower gum | pT4a | pN3b | M0 | Yes |
| OSCC 19 | F | 75 | Cheek | RpT1 | pN0 | M0 | Yes |
| OSCC 20 | F | 70 | Tongue | pT2 | pN3b | M0 | Yes |
| OSCC 21 | F | 72 | Tongue | pT3 | pN2c | M0 | No |
| OSCC 22 | F | 85 | Tongue | pT3 | pNo | M0 | No |

Supplementary Table 2

| **Patient** | **Sex** | **Age at surgery** | **Localisation** | **pTNM classification** | | | **Recurrence Yes/No** |
| --- | --- | --- | --- | --- | --- | --- | --- |
|  |  |  |  | **pT-stage** | **pN-stage** | **M-stage** |  |
| OSCC 1 | F | 78 | Lower gum | pT1 | pN0itc | M0 | No |
| OSCC 2 | M | 72 | Tongue | pT3 | pN2c | M0 | Yes |
| OSCC 3 | M | 55 | Tongue | pT3 | pN3b | M0 | No |
| OSCC 4 | M | 57 | Tongue | pT3 | pN0 | M0 | No |
| OSCC 5 | F | 68 | Lower gum | pT4a | pN0 | M0 | No |
| OSCC 6 | M | 61 | Tongue | pT1 | pN1 | M0 | No |
| OSCC 7 | F | 64 | Lower gum | pT4a | pN3b | M0 | Yes |
| OSCC 8 | M | 71 | Upper gum | T4a | pN0 | M0 | Yes |
| OSCC 9 | F | 71 | Cheek | pT2 | pN0 | M0 | No |
| OSCC 10 | F | 76 | Tongue | pT2 | pN2b | M0 | No |
| OSCC 11 | M | 52 | Tongue | pT2 | pN0 | M0 | No |
| OSCC 12 | M | 40 | Tongue | pT1 | pN0 | M0 | No |
| OSCC 13 | F | 57 | Tongue | pT2 | pN1 | M0 | No |
| OSCC 14 | M | 67 | Floor of the mouth | pT1mi | pN0 | M0 | No |
| OSCC 15 | F | 60 | Tongue | pT2 | pN0itc | M0 | No |
| OSCC 16 | F | 59 | Tongue | pT2 | pN0 | M0 | No |
| OSCC 17 | F | 87 | Floor of the mouth | pT2 | pN1 | M0 | No |
| OSCC 18 | F | 61 | Lower gum | pT4a | pN3b | M0 | Yes |
| OSCC 19 | F | 75 | Cheek | RpT1 | pN0 | M0 | Yes |
| OSCC 20 | F | 70 | Tongue | pT2 | pN3b | M0 | Yes |
| OSCC 21 | F | 72 | Tongue | pT3 | pN2c | M0 | No |
| OSCC 22 | F | 85 | Tongue | pT3 | pNo | M0 | No |

. List of antibodies, commercial kits and reagents from different providers used in the study.

| **Antibody** | **Provider** | **Cat. Number** | **Fluorochrome** | **Clone** |
| --- | --- | --- | --- | --- |
| CD3 (FACs) | BD Biosciences | 612940 | BUV496 | UCHT1 |
| CD4 (FACs) | BD Biosciences | 562970 | BV510 | SK3 |
| CD8 (FACs) | BD Biosciences | 555634 | FITC | HIT8a |
| Siglec 3 (FACs) | BD Biosciences | 742015 | BUV85 | WM53 |
| Siglec 5 (FACs) | BD Biosciences | 749693 | BV786 | 1A5/CD170 |
| Siglec 7 (FACs) | BD Biosciences | 558372 | PE | F023-420 |
| Siglec 9 (FACs) | BD Biosciences | 569423 | APC | K8 |
| Siglec 10 (FACs) | BD Biosciences | 566588 | BV421 | 5G6 |
| CD4 (IF) | Cell Signaling Technology | 48274 | - | EP204 |
| CD8α (IF) | Cell Signaling Technology | 85336 | - | D8A8Y |
| Siglec-3 (IF) | Abcam | ab269456 | - | EPR23051-101 |
| Siglec-5 (IF) | Abcam | ab307434 | - | EPR266250 |
| **Commercial kits** | | | | |
| Tumour Dissosiation, Human | Miltenyi Biotec | #130-095-929 | - | - |
| Novolink Polymer Detection Kit | leica | RE7140-K | - | - |
| VectaPlex Antibody Removal Kit | Vector Labs | VRK-1000 | - | - |
| TSA reagents: CF488  CF568  CF647 | Biotium | 92173  99832  99823 | - | - |
| **Other** | | | | |
| DMEM | Gibco | #21331020 | - | - |
| MACS Tissue Storage | Miltenyi Biotec | #130-100-008 |  |  |
| Gentle Macs C tubes | Miltenyi Biotec | #130-096-334 |  |  |
| FC-Block | BD Biosciences | #564220 |  |  |
| PBS | Gibco | #2812-019 |  |  |
| Briliant stain buffer | BD Biosciences | #563794 |  |  |
| Compbeads | BD Biosciences | #552843 |  |  |
| CS&T beads | BD Biosciences | #650621 |  |  |
| Cellstrainer (100µm) | BD Biosciences | #352360 |  |  |
| FBS | Thermo Fisher scientific | #10270106 |  |  |
| HistoClear | National Diagnostics | - | - | - |
| Leica BOND Primary Antibody Diluent | Leica Biosystems | AR9352 | - | - |
